# Internet use and rural-urban mental health inequalities: Evidence from China

**DOI:** 10.3389/fpubh.2023.1107146

**Published:** 2023-01-26

**Authors:** Weihao Nie, Mingzheng Hu, Xin Ye

**Affiliations:** ^1^Faculty of Arts and Humanities, Philosophy, Politics and Economics of Health, University College London, London, United Kingdom; ^2^China Center for Health Development Studies, Peking University, Beijing, China; ^3^Institute for Global Public Policy, LSE-Fudan Research Centre for Global Public Policy, Fudan University, Shanghai, China

**Keywords:** urban-rural differences, Internet use, mental health status, mental health inequalities, China

## Abstract

**Introduction:**

In the context of the new digital era, clarifying the relationship between Internet use and urban and rural residents' mental health is of important value for reducing rural-urban health inequalities. This paper aims to study the association between Internet use and rural-urban mental health inequalities.

**Methods:**

Based on the data of the China Family Panel Studies (CFPS) in 2020, we firstly examined the existence and specific manifestation of mental health inequalities between urban and rural residents. Secondly, we examined the mediating effect of Internet use by the Bootstrap mediating effect measure. Finally, we verified the robustness of the mediating effect.

**Results:**

There are significant mental health inequalities between urban and rural residents, and urban residents have better mental health than rural residents (*p* < 0.01). In addition, the test results for the mediating effect of Internet use on mental health inequalities between urban and rural residents were significant (*p* < 0.01), with a direct effect of −0.028 (*p* < 0.01) and an indirect effect of −0.49 (*p* < 0.01), and this result remained significant in the robustness test.

**Discussion:**

In such a new age of the Internet, mental health inequalities between urban and rural residents objectively did exist, and the use of the internet played a positive mediation effect on the formation of mental health inequalities between urban and rural areas.

## 1. Introduction

In recent years, the Internet is embedded in social life, which is like a double-edged sword. On the one hand, online fraud, online pornography, online violence, and other functions endanger people's lives; On the other hand, online medical, virtual social and other Internet features can facilitate the healthy life of residents. Currently, although the Internet has been promoted nationwide in China, Internet users in China are still mainly urban residents, with relatively low Internet penetration in rural areas. There is a significant difference in the number of Internet users between urban and rural areas. According to China Internet Network Information Center (CNNIC), as of the end of March 2020, the total number of Internet users reached 904 million, of which 255 million were in rural areas and the Internet penetration rate was 46.2%. In contrast, 649 million were in urban areas and the Internet penetration rate was as high as 76.5%, with a significant gap of 30.3% between the two.

At the same time, Chinese society has long been characterized by health inequalities between urban and rural residents (1–3), especially in mental health (4, 5). A meta-analysis showed that the prevalence of depressive symptoms was nearly 10% higher in rural areas than in urban areas (6). In addition, several cross-sectional studies have shown that there is an urban-rural gap in mental health, especially among older adults and women (7, 8).

Under such background, we cannot help but think that, since there are differences between urban and rural areas in both internet use and mental health, do urban-rural differences affect the mental health inequalities of the population through Internet use? We focused on urban-rural differences because the internet coverage could be different in urban and rural areas. Firstly, due to the economic factors, richer towns/villages may have good internet facilities compared to those in poorer towns/villages (9, 10). Secondly, many of the younger generations living in urban areas are probably using the Internet. But older people in rural areas may be mostly less likely to be online use (11, 12).

**Figure 1:**
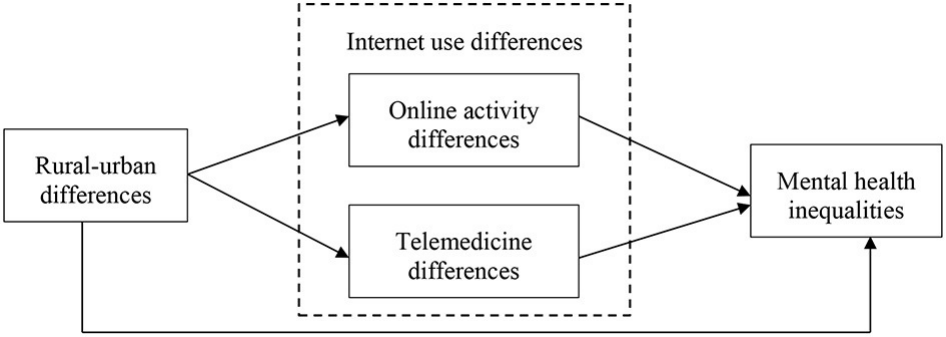
Theoretical framework of internet use and rural-urban mental health inequalities.

A potential theoretical framework for the impact of Internet use on rural-urban mental health inequalities is shown in Figure 1. Differences in Internet use between urban and rural residents may affect rural-urban mental health inequalities in two ways. Firstly, Internet users generally have more opportunities for social engagement, social activities, and recreation (13, 14), thus providing depressed and lonely individuals with more opportunities for interpersonal and emotional communication, which is beneficial to mental health. Secondly, the use of the Internet can provide healthcare services through telemedicine (15) and facilitates the exchange of medical knowledge (16), thereby treating diseases more conveniently.

There have been studies with samples from developed countries showing that Internet use can cause urban-rural health inequalities (16), but these studies focus on inequalities between urban-rural physical health and ignore inequalities in mental health. In addition, these findings from developed countries may not apply to developing countries. Because developed countries have well-established healthcare systems and high urbanization rates, while rural patients in developing countries do not have access to the same healthcare resources. Therefore, there is a need to explore this issue separately in developing countries. China, as the largest developing country in the world, provides a good sample for exploring this issue. In China, although there have been numerous studies (17–22) demonstrating the role of mediating variables between urban and rural health inequalities such as medical accessibility (19) and socioeconomic status (20, 21), the mediating mechanism of the internet use has not received much attention.

Therefore, based on the above research background, we attempt to choose the latest released China Family Panel Studies (CFPS) data in China in 2020 to study the following issues: first, in the context of the new digital era, we demonstrate the objective existence of health inequalities between urban and rural residents in China and their specific manifestations in mental health. Second, using the world's largest developing country as a sample, we examine the mediating effects of Internet use in rural-urban health inequalities, so as to provide experiences for developing countries in eliminating rural-urban health inequalities.

## 2. Materials and methods

### 2.1. Data sources

The data used in this study are from the newly released 2020 China Family Panel Studies (CFPS) individual database. CFPS data is a nationally representative longitudinal study of Chinese communities, families, and individuals. Since 2010, CFPS data has been surveyed every 2 years, covering 25 provinces/regions or their administrative levels (i.e., municipalities and autonomous regions) out of 31 provinces/regions in China (23). For the CFPS in 2010, the multi-stage probability distribution was used to stratify the samples, and five provinces/regions (Gansu, Guangdong, Henan, Liaoning, and Shanghai) were selected for preliminary oversampling (1,600 families in each province/region, or 8,000 families in total) to obtain regional comparison, and another 8,000 families were weighted from other provinces/regions, making the entire CFPS sample nationally representative. CFPS has been approved by the Biomedical Ethics Review Committee of Peking University (ID: IRB00001052-14010). We chose CFPS as the data source for this study because of its broader research agenda, wider population coverage, and its national representation. For the original sample of 28,590, we removed samples (*N* = 4,065) with the following five conditions in the selected variables: unable to judge, missing, not applicable, refused to answer, and don't know, thus deriving the final sample size of 20,536.

### 2.2. Measures

#### 2.2.1. Dependent variable: Mental health

Depression is a commonly used variable to represent mental health (24–26). In this study, we also used depressive symptoms to measure mental health and used the CES-D8 scale to assess the severity and frequency of certain feelings and behaviors. Respondents were asked how often they felt unable to move forward in life, sad, happy, lonely, happy, poor sleep, hard to do things, and depressed. The scores for these items range from 1 (no time) to 4 (all or almost all of the time). We add the eight items together to develop a mental health index. The higher the score, the unhealthier the individual's psychology is.

In addition, we conducted robustness tests using subjective wellbeing. In psychology, the concepts of happiness, wellbeing, and mental health are often used as synonyms (27). The World Health Organization defines mental health as a state of well-being in which individuals are able to reach their potential, cope with the stresses of life, work productively, and contribute to society (28). Therefore, it is increasingly recognized that both mental health (e.g., depression) and wellbeing should be considered when measuring mental health (29). Mental health cannot be separated from subjective wellbeing, which is a positive aspect of mental health (30). Hence, subjective wellbeing was used for robustness testing in this study. We used the question “How happy do you think you are” from the CFPS to measure subjective wellbeing.

#### 2.2.2. Core independent variable: Household registration status

Based on China's household registration system, we have divided people into urban and rural residents. We define “rural” as the respondents who report that they currently have agricultural household registration and “urban” as those who report that they have non-agricultural household registration or urban resident household registration (31). The household registration status is assigned a value of 1 if the respondent is an urban resident, and 0 if the respondent is a rural resident.

#### 2.2.3. Mediating variable: Internet use

In this paper, Internet use refers to the behavior of people who can use Internet technology for learning, socializing, and entertainment through modern information and communication technology such as cell phones and computers. The variable “whether or not to access the Internet by computer” was generated by the questionnaire, and the answer “yes” was assigned a value of 1, and the answer “no” was assigned a value of 0.

#### 2.2.4. Control variables

The control variables include age, gender, marriage, education, income level, and work status. Among them, age, gender, and marriage can be categorized as natural attributes affecting health status, while education, income level, and work status are mainly indicators of socioeconomic status. Previous studies have shown that as age increases, the health status of the population becomes worse (32, 33); gender is related to age (34, 35); the lower level of education, the worse health status (36, 37); marital status also affects the emotional health of the population (38, 39); the higher income, the better health (40); work status has also been found to be strongly associated with health (41). Therefore, in this paper, the above variables that may affect health were controlled to exclude relevant interference. In terms of assignment, age is a continuous variable, ranging from 9 to 104 years old; gender is assigned as 1 for male and 0 for female; education is differentiated according to education level, ranging from illiterate to Ph.D., respectively, on a scale of 1–8; and marriage is divided into two types: married and unmarried, with 1 indicating married. Income level is the respondent's score of their income in the local position, from high to low 1–5. Work status is classified as having a job or not, with a value of 1 assigned to having a job and 0 to not having a job.

### 2.3. Statistical analysis

This study first verifies the effect of urban-rural disparities on the mental health of the population, and the OLS regression model is shown in Equation (1):


(1) $$health_{i}\;=\;\alpha_{0}\;+\;\alpha_{1}urban_{i}+\delta_{1c}X_{i}+\varepsilon_{1i}$$

Where *health*_*i*_ is the explanatory variable, which contains mental health. *Urban*_*i*_ is the core independent variable. *X*_*i*_ is a set of control variables. The estimated coefficient α_1_ is the coefficient of the urban-rural effect on mental health, which determines the existence of urban-rural mental health inequalities according to whether it is significant or not. The positive or negative of α_1_ determines the specific manifestation of urban-rural mental health inequalities. ε_*i*_ is a random disturbance term.

In addition, Equations (1)–(3) is the mediating effect model developed in this paper, where *Internet*_*i*_ is the mediating variable representing whether or not to use the Internet. To test the mediating effect of Internet use, we choose the bootstrap method. The bootstrap method uses the study sample as the overall sample, and repeatedly draws a certain number of samples from the study sample by means of put-back sampling, and takes the mean value of the parameters obtained from each sample as the final estimation result. This method has high statistical validity and can make the parameter estimation of the model more accurate (42). In this paper, the Bootstrap mediation test with 500 repetitions of sampling was conducted using stata16 software.


(2) $$Internet_{i}\;=\;\beta_{0}\;+\;\beta_{1}urban_{i}+\delta_{2c}X_{i}+\varepsilon_{2i}$$

(3) $$health_{i}\;=\;\gamma_{0}\;+\;\gamma_{1}urban_{i}+=\;\gamma_{2}internet_{i}\;+\;\delta_{3c}X_{i}+\varepsilon_{3i}$$

## 3. Results

### 3.1. Descriptive results

According to the results in Table 1, the mean value of mental health is 13.439, indicating that the majority of respondents are not so depressed; the mean value of household registration status is 0.281, indicating that 28.1% of the respondents are urban households; the mean value of the Internet is 0.213, representing only 21.3% of the respondents use the Internet, which indicates that the penetration rate of the Internet in China is not so high; the mean age of the interviewees is 44.263, indicating that the majority of respondents are middle-aged. In addition, 50.5% of the respondents are male, 43.4% are married and 78.2% are having work. There are no outliers in the sample.

**Table 1:** Descriptive statistics of the variables.

	**N**	**Mean**	**SD**	**Min**	**Max**
Mental health	24,525.000	13.439	4.048	8.000	32.000
Urban household registration	22,948.000	0.281	0.450	0.000	1.000
Internet	24,904.000	0.213	0.409	0.000	1.000
Age	28,590.000	44.263	19.467	9.000	104.000
Gender	25,114.000	0.505	0.500	0.000	1.000
Marriage	23,048.000	0.7651	0.424	0.000	1.000
Income level	21,238.000	2.929	1.050	1.000	5.000
Edu	28,504.000	2.783	1.460	1.000	8.000
Work stage	22,932.000	0.782	0.413	0.000	1.000

### 3.2. Analysis of mental health inequalities between urban and rural residents

As shown in column (1) of Table 2, without control variables, the results of the baseline regression show the objective existence of mental health inequalities between urban and rural areas, with a regression coefficient of −0.912, which is significant at the 1% level, showing that urban respondents have better mental health than rural respondents. Besides, with control variables in the model, as shown in column (1) of Table 2, the results are still significant.

**Table 2:** Regression results.

	**(1)**	**(2)**
**Variables**	**Mental health**	**Mental health**
Urban household registration	−0.912^***^	−0.484^***^
	(0.060)	(0.069)
Control	No	Yes
_cons	13.797^***^	17.776^***^
	(0.032)	(0.183)
N	22,384.000	20,536.000
F	229.390	187.930

^***^p < 0.01.

### 3.3. Analysis of the mediating effect of internet use

To further verify the mediating role of Internet use, this paper uses the bootstrap mediating effect measure to decompose the impact (Table 3). The estimated coefficient of direct effect is −0.49, and the estimated coefficient of indirect effect is −0.028, and all the two effects pass the 5% significance test. All coefficients are negative, indicating that urban-rural differences could affect respondents' mental health not only directly, but also indirectly through Internet use, indicating that the respondents could significantly improve their health status through the Internet.

**Table 3:** Decomposition of the effect of urban and rural areas on mental health.

	**Coefficient**	**Std. err**.	***p* > z**
Indirect effects	−0.028^***^	0.009	0.009
Direct effect	−0.490^***^	0.066	0.000

^***^p < 0.01.

### 3.4. Robustness test

We used three methods for robustness testing. Firstly, using CFPS 2020 data, we conducted a selection test on the sample by removing a portion of the residents with the lowest depression score from the analyzed sample to test the mediating effect of Internet use in the remaining sample. As shown in column (1) of Table 4, the results show that the above study findings still hold. Secondly, using CFPS 2020 data, we used subjective wellbeing as a replacement variable, as shown in column (2) of Table 4, and the mediating effect test remains significant. Third, to enhance the causal inference validity, we used the CFPS longitudinal survey data from 2010 to 2020. As shown in columns (3) and (4) of Table 4, the mediating effect test results were significant for either depression or subjective wellbeing as a measure of mental health.

**Table 4:** Robustness test result.

	**CFPS (2020)**		**CFPS (2010–2020)**	
	**(1)**	**(2)**	**(3)**	**(4)**
	**Depression** **(unremoved sample)**	**Subjective** **wellbeing**	**Depression**	**Subjective wellbeing**
Indirect effects	−0.031^***^	0.014^**^	−0.025^***^	0.003^***^
	(0.012)	(0.006)	(0.005)	(0.0003)
Direct effect	−0.427^***^	0.134^***^	−0.682^***^	0.021^***^
	(0.066)	(0.031)	(0.032)	(0.002)

^**^p < 0.05, ^***^p < 0.01.

## 4. Discussion

This paper examined the current state of urban-rural mental health inequalities using regression analysis and analyzed the mediating role of Internet usage by Bootstrap mediating effect measure, and the following conclusions were drawn.

First, mental health inequalities between urban and rural residents exist objectively, mainly manifested by the fact that urban respondents have better mental health than rural respondents. The social-ecological system theory (43) suggests that individual health is influenced by many factors such as interpersonal, organizational, community, public policies, and social environment. Rural and urban residents differ in many ways, including education, income, organization, interpersonal, and living communities. People living in rural areas travel farther to receive care, they are less likely to have access to quality health care and visit healthcare providers frequently, and therefore have poorer health status. This is consistent with social-ecological systems theory and with the findings of other scholars (3).

Second, Internet use plays a partially mediating role in the formation of health inequalities between rural and urban residents, and the effect is tested by Bootstrap methods. This finding can be explained in two ways: first, in terms of the interpersonal affective aspects, Internet users will have more opportunities for social participation, social activities, and recreation (13, 14), which is beneficial to mental health. Second, in terms of information acquisition, the Internet is an important channel for people to obtain health information, and Internet users can use the Internet to acquire health knowledge, search for information on diseases, enhance health prevention and care, participate in online health activities, and improve their lifestyles to improve their health (44). The Internet is an important channel for people to obtain health information.

There are certain limitations in this study. There may be a reverse causal relationship between Internet use and residents' mental health. For example, loneliness may enhance residents' use of the Internet. But in this study, we measured mental health based on residents' level of depression in the past week, so this health indicator is immediate, whereas Internet use refers to Internet use “in the past year.” Thus, there is a time lag between the respondents' mental health and Internet use variables, which may mitigate the possible endogeneity risk to some extent. However, although the potential endogeneity risk is relatively small, this does not completely address or avoid the possible endogeneity risk. Future studies can select other methods to better solve this problem. Despite the limitations, this work also has several strengths. Firstly, there are few studies on the relationship between Internet use and rural-urban mental health inequality, and the limited studies mainly take developed countries as samples, lacking research on developing countries. Secondly, this paper takes China, the largest developing country in the world, as a sample for research, which can provide experience for developing countries to eliminate rural-urban mental health inequality. Thirdly, most of the data selected in the existing research on this topic in China are not timely. In the new era of the Internet, this paper uses the latest CFPS 2020 data to test the objective existence and specific manifestations of rural-urban mental health inequality, therefore providing the latest empirical evidence in China.

## 5. Conclusion

This paper examined the current status of mental health inequalities between urban and rural residents using regression analysis and analyzed the mediating role of Internet use by the Bootstrap method. The results showed that mental health inequalities exist between urban and rural areas, and Internet use plays a mediating effect in it. Rural-urban health inequalities are an important topic for many countries around the world, and with the advent of the digital age, the use of the Internet provides new perspectives to explain rural-urban health inequalities. It is suggested that additional research on how increasing Internet access affects health in rural and urban areas is needed in the future.

## Data availability statement

The original contributions presented in the study are included in the article/supplementary material, further inquiries can be directed to the corresponding author.

## Ethics statement

The studies involving human participants were reviewed and approved by the Biomedical Ethics Review Committee of Peking University (ID: IRB00001052-14010). Written informed consent to participate in this study was provided by the participants' legal guardian/next of kin.

## Author contributions

Conceptualization, data curation, writing, and writing—original draft: WN and MH. Methodology and writing—review and editing: WN, MH, and XY. Validation and supervision: XY. Formal analysis: WN and MH. All authors have read and agreed to the published version of the manuscript.
